# Supplementary material for: Content-rich biological network constructed by mining PubMed abstracts
Source: BMC Bioinformatics. 2004 Oct 8;5:147. doi: 10.1186/1471-2105-5-147 (PMC528731; doi:10.1186/1471-2105-5-147)
Supplement: Additional File 2 — The original results of the above study (non-essential files are deleted to keep the file size under the limit set by BMC bioinformatics). [file 1471-2105-5-147-S2.bz2 › chilibotAdditionalFile2/dip10/478ID7592830E10564/html/FY_CXCL1.html]

 


 **FY** and **CXCL1** 
  
Found 10 abstracts in PubMed, retrieved 10.  
 

 What does Google say? 
 PDF only 
| .edu only 

---

**Interactive relationship** (e.g. stimulation, inhibition, etc)

**Neutral relationship**- Receptor binding and biological studies with the alanine scan mutants of MGSA  [ **CXCL1** ]  demonstrate that MGSA  [ **CXCL1** ]  binds to DARC  [ **FY** ]  and the IL 8RB through distinct binding regions.  Ref: 7744785 J Biol Chem, 1995
- The hybrid receptor also bound RANTES, MCP 1, and MGSA  [ **CXCL1** ]  E6A which binds DARC  [ **FY** ] , but not IL 8RB, but not MIP 1 alpha, similarly to DARC  [ **FY** ] .  Ref: 7592830 J Biol Chem, 1995
- Biopanning of phage peptide libraries with an anti Fy6 monoclonal antibody led to the identification of the motif Phe22 Glu23, the mutation of which altered the binding of both anti Fy6 and chemokines interleukin 8, MGSA  [ **CXCL1** ] , RANTES regulated on activation normal T cell expressed to DARC  [ **FY** ]  transfectants.  Ref: 9195930 J Biol Chem, 1997
- Scatchard analysis demonstrated that a chimeric DARC  [ **FY** ]  receptor, composed of the E1 domain of DARC  [ **FY** ]  and the predicted hydrophobic helices and loops of interleukin 8RB IL 8RB, bound IL 8, and MGSA  [ **CXCL1** ]  with KD values almost identical to the wild type receptors and bound a repertoire of C X C and C C chemokines characteristic of DARC  [ **FY** ] .  Ref: 8558064 J Leukoc Biol, 1996
- Alanine scanning mutagenesis of the charged amino acids of melanoma growth stimulating activity MGSA  [ **CXCL1** ]  was used to identify specific residues that are involved in binding to the human erythrocyte Duffy antigen chemokine receptor  [ **FY** ]  DARC  [ **FY** ]  and to the type B interleukin 8 receptor IL 8RB on neutrophils.  Ref: 7744785 J Biol Chem, 1995

**Non-interactive relationship** (e.g. studied together, co-existance, homology, etc.)

- Several lines of evidence in the present study indicate that the Duffy blood group  [ **FY** ]  antigen is the erythrocyte receptor for the chemokines interleukin 8 IL 8 and melanoma growth stimulatory activity  [ **CXCL1** ]  MGSA  [ **CXCL1** ] .  Ref: 7689250 Science, 1993
